# Adlay Seed (*Coix lacryma-jobi* L.) Extracts Exhibit a Prophylactic Effect on Diet-Induced Metabolic Dysfunction and Nonalcoholic Fatty Liver Disease in Mice

**DOI:** 10.1155/2020/9519625

**Published:** 2020-05-29

**Authors:** Hao Chiang, Hsu-Feng Lu, Jui-Chieh Chen, Yu-Hsin Chen, Hsi-Tai Sun, Hsiu-Chen Huang, Hsiao-Hsuan Tien, Cheng Huang

**Affiliations:** ^1^Department of Biotechnology and Laboratory Science in Medicine, National Yang-Ming University, Taipei 11221, Taiwan; ^2^Departments of Clinical Pathology, Cheng Hsin General Hospital, Taipei 11221, Taiwan; ^3^Department of Restaurant, Hotel and Institutional Management, Fu-Jen Catholic University, New Taipei 24205, Taiwan; ^4^Department of Biochemical Science and Technology, National Chiayi University, Chiayi 60004, Taiwan; ^5^Taichung District Agricultural Research and Extension Station, Council of Agriculture, Changhua County 51544, Taiwan; ^6^A.T.P. Co., Ltd., Taipei 10452, Taiwan; ^7^Department of Applied Science, National Tsing Hua University South Campus, Hsinchu 30014, Taiwan; ^8^Department of Earth and Life Sciences, University of Taipei, Taipei 11153, Taiwan

## Abstract

Nonalcoholic fatty liver disease (NAFLD) is common worldwide and closely associated with metabolic dysfunction. NAFLD leads to a higher risk of development of severe liver diseases, such as nonalcoholic steatohepatitis (NASH), liver cirrhosis, and hepatocellular carcinoma (HCC). To date, no pharmacotherapy targeting NAFLD has received general approval. Adlay is a plant that has been used as traditional herbal medicine in Asia and is a promising candidate to solve this global issue. We have established a mouse model of NAFLD by feeding a high-fat diet (HFD) for 10 weeks. Here, ethanolic or water extracts of adlay seed (ASE and ASW, respectively), mixed with HFD, were fed to the mice for 10 weeks. The ASE and ASW treatment ameliorated hyperglycemia and improved the glucose tolerance and insulin resistance in the HFD mice. Hyperlipidemia in HFD mice was prevented by the ASE and ASW diet. In addition, the ASE and ASW supplementation attenuated hepatic steatosis and inflammation, improved liver function, and caused no harm to the kidneys. Moreover, the mechanism of the effect of ASE and ASW on inhibiting hepatic lipogenesis and inducing fatty acid *β*-oxidation was certified by the simulated human fatty liver cell model. Our study showed the regulatory potential of the extracts of adlay seeds for alleviating NAFLD, as well as related liver and metabolic diseases.

## 1. Introduction

The liver is a vital organ that plays a major role in the metabolic processes of carbohydrates, proteins, and lipids, and a properly functioning liver is essential to health. Liver diseases caused by metabolic dysfunction lead to serious health problems. Nonalcoholic fatty liver disease (NAFLD), resulting from metabolic disorders, is considered to be a leading cause of abnormal liver function [1, 2]. Several studies have indicated that NAFLD may develop into the end stage of liver disease and HCC; it can no longer be regarded as a trivial disease but is a risk factor for serious liver disease [3, 4]. The global prevalence of NAFLD in the general population has been estimated to be about 30% and has doubled in the past two decades [5, 6], a major concern for human health.

Insulin resistance, resulting from long-term, sustained hyperglycemia, leading to impaired insulin-stimulated glucose utilization and glycogen synthesis, is the key pathogenic feature of metabolic syndrome and is now regarded as the most common risk factor for the development and progression of nonalcoholic fatty liver disease (NAFLD) [7]. The pathophysiology of NAFLD is induced by multiple factors [8], such as obesity, insulin resistance [9], and dysregulation of lipid metabolism [10], which also interact with each other in a dynamic manner. Until now, no pharmacotherapy or treatment guideline has been approved for the treatment of NAFLD [11], emphasizing the need for development of novel treatments for NAFLD.

Adlay (*Coix lacryma-jobi* L.), also called Jobs tears or Chinese pearl barley, is a medicinal plant which has been grown widely in Taiwan, China, and Japan. Adlay has been served as nourishing food and used in traditional Chinese medicine for many years for the treatment with inflammatory diseases, warts, neuralgia, and neoplastic diseases [12]. A number of studies have shown several physiological effects of adlay and its biologically active components on various parts of the plant [13]. Adlay seeds are the major medicinal part and contain a range of bioactive ingredients, such as polysaccharides, coixol, proteins, lipids, and polyphenols [14]. Several studies have demonstrated that adlay seeds have anti-inflammatory activity [12], hypoglycemic activity [15], the ability to decrease the amount of lipid components in the serum [16], hypocholesterolemic activity [17], and other beneficial effects on humans [18]. However, as far as we know, little is known about the effect of supplementation with extracts of adlay seeds on dietary fat-induced metabolic syndromes and NAFLD.

In this study, we examined the effect of ethanolic and water extracts of adlay seeds on high-fat diet- (HFD-) induced metabolic dysregulation, the status of NAFLD, and the inflammation of liver tissues *in vivo*. In our HFD mouse model, the animals showed abnormal metabolism of lipid and glucose, as well as significant dyslipidemia and markers of hepatic steatosis. The adlay seed extracts improved glucose tolerance and prevented dyslipidemia and HFD-induced NAFLD in the mice. Moreover, we utilized a human fatty liver cell model by using human immortalized primary hepatocyte, HuS-E/2 cells, to investigate the mechanism of adlay seed extracts on improving NAFLD. Our findings provide evidence for the use of supplements of adlay seed extracts for the prevention of dietary fat-induced metabolic syndrome and NAFLD.

## 2. Materials and Methods

### 2.1. Preparation of Adlay Seed Extracts

Adlay plants (*Coix lacryma-jobi* L. var. ma-yuen Stapf) were grown in the Taichung District Agricultural Research and Extension Station, Taichung, Taiwan. Whole adlay grains were grinded into 20 mesh powder and extracted with 70% ethanol. The ethanolic extract of adlay seeds (ASE; CoiXtreme™, A.T.P. CO., LTD., Taiwan) was concentrated and particulated with lactose powder and using coixol as an indicator compound at a concentration of 620 ppm. Water extracts of adlay seeds (ASW) were generated by grinding the whole adlay grains into 20 mesh powder and extracting with water using an ultrasonic bath for 30 minutes at 50°C (Branson Co.). The extracts were characterized as water-soluble polysaccharides and then concentrated, oven dried at 50°C, and grinded into powder. Then, the ASE and ASW were stored at −20°C as functional materials.

### 2.2. Animals

Five-week-old male C57BL/6J mice were purchased from the National Laboratory Animal Center, Taiwan, and maintained in a temperature-controlled room on a 12 h light-dark cycle at the Animal Center of the National Yang-Ming University, Taiwan. They were housed and had free access to food and drinking water. Mice fed with a standard diet and adapted to the environment for 1 week were subsequently divided randomly into four groups. The ND group (*n* = 6) continued on the normal diet, whereas the other three groups (*n* = 6 per group) were switched to the high-fat diet (HFD group, 494 kcal/100 g, 45% energy as fat; TestDiet Inc., USA), the HFD mixed with a 1% (weight for weight) ethanolic extract of adlay seeds (1% ASE group), and the HFD mixed with a 3% (weight for weight) water extract of adlay seeds (3% ASW group) for 10 weeks. Food consumption and weight gain were measured daily and weekly, respectively. All mice were sacrificed at the end of the experimental period. Serum samples, liver tissue, and epididymis adipose tissue were harvested for further analysis. The experimental protocol was approved by the Animal Research Committee of the National Yang-Ming University (IACUC no. 1070213), and all procedures followed the Guide for the Care and Use of Laboratory Animals (NIH publication, 85-23, revised 1996) and the guidelines of the Animal Welfare Act, Taiwan.

### 2.3. Blood Glucose, Serum Insulin, Intraperitoneal Glucose Tolerance Test, and Homeostasis Model Assessment of Insulin Resistance Index

The analysis of blood glucose, serum insulin, intraperitoneal glucose tolerance test, and the homeostasis model assessment of insulin resistance index were performed as described previously [19].

### 2.4. Biochemical Characterization

Serum triglyceride (TG), total cholesterol (TC), high-density lipoprotein cholesterol (HDLC), glutamic oxaloacetic transaminase (GOT), glutamic pyruvic transaminase (GPT), creatinine (CRE), and blood urea nitrogen (BUN) were measured using enzymatic assay kits with a FUJI DRI-CHEM analyzer (Fujifilm, Tokyo, Japan). The low-density lipoprotein-cholesterol (LDLC) level was calculated as TC − (HDLC + TG/5) [20].

### 2.5. Triglyceride and Cholesterol Analysis of Liver Tissue

For hepatic triglyceride and cholesterol determinations, the methods were performed as described previously [19].

### 2.6. Cell Line

HuS-E/2 cells, kindly provided by Kunitada Shimotohno (Kyoto University, Japan), were grown as described previously [21]. To generate fatty liver disease cell model, HuS-E/2 cells at 70% confluence were incubated with 0.1 mM oleic acid (OA) for 18 h.

### 2.7. Antibodies and Western Blot Analysis

Antibodies against AMPK, ACC, pACC (Ser79), and tubulin were obtained from GeneTex. The anti-pAMPK (Thr172) antibodies were from Cell Signaling. The horseradish peroxidase-conjugated anti-mouse or anti-rabbit IgG antibodies were from Abcam. Western blot analysis was performed as previously described [19].

### 2.8. Quantitative Real-Time Polymerase Chain Reaction

In brief, total mRNA of mice liver tissues or HuS-E/2 cells were extracted from TRZOL reagent and reverse transcribed into cDNA by using a Deoxy RT kit (Yeastern Biotech.). All qPCR reactions were performed with SYBR Green PCR Master Mix (Applied Biosystems). The fluorescent signal data were processed using StepOne software. The primers used were described previously [19].

### 2.9. Statistical Analysis

Data obtained from all experiments are shown as means ± SEM. All the differences were assessed for significance using the one-way ANOVA followed by Dunnett's honest significant difference post hoc tests. Asterisks indicate that the values differed significantly from the control (^*∗*^*p* < 0.05; ^*∗∗*^*p* < 0.01; ^*∗∗∗*^*p* < 0.001; ^#^*p* < 0.05; ^##^*p* < 0.01; and ^###^*p* < 0.001).

## 3. Results

### 3.1. ASE and ASW Improved Glucose Hemostasis in HFD Mice

**Figure 1:**
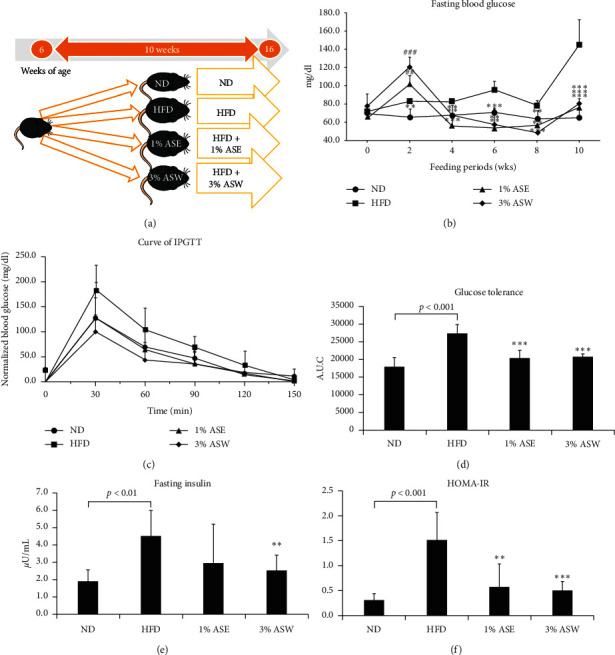
Effect of ASE and ASW on glucose metabolism, glucose tolerance, and insulin resistance in C57BL/6J mice fed by HFD. (a) The experimental approach. Six-week-old male C57BL/6 mice were divided into four groups and fed normal diet (ND), high-fat diet (HFD), 1% ASE mixed with HFD, and 3% ASW mixed with HFD for 10 weeks. At 16 weeks of age, the follow-up analysis was conducted. (b) Blood glucose levels after 16 h of fasting. (c) Curve of normalized IPGTT. Data are normalized by subtracting the baseline value (fasting blood glucose) from the measured value in each mouse. (d) Area under the curve (AUC) of blood glucose in the IPGTT assay. (e) Serum insulin levels after 16 h of fasting. (f) The HOMA-IR index calculated using fasting blood glucose and insulin levels. Data are shown as means ± SEM (*n* = 6 in each group). HFD vs. ND, ASE, or ASW, ^###^*p* < 0.001 in increased level. HFD vs. ND, ASE, or ASW, ^*∗∗*^*p* < 0.01; ^*∗∗∗*^*p* < 0.001, in decreased level.

To determine the effect of ASE and ASW on NAFLD, we used an established HFD-induced mouse model of NAFLD, produced by feeding an HFD, and the HFD mice were treated with ASE or ASW for 10 weeks (Figure 1(a)). The body weight gain and the adipose tissue weight of the HFD group were significantly greater than the ND group (Table 1). This suggests that the HFD mice had a tendency to develop metabolic complications, which fit the main characteristics of central obesity. The amount of food consumed by the mice did not differ significantly among the groups. Interestingly, the liver weights of 3% ASW mice were significantly lower than those of HFD mice after 10 weeks of diet (Table 1).

Moreover, we found that continuous HFD feeding for 10 weeks led to a significant increase in fasting blood glucose, area under the curve (AUC) of IPGTT, and fasting insulin in HFD mice (Figures 1(b)to 1(e)), suggesting glucose tolerance and insulin sensitivity were impaired. The calculated HOMA-IR (Figure 1(f)) confirmed that insulin resistance was induced by feeding an HFD. Treatment with ASE and ASW significantly ameliorated not only the increased levels of fasting glucose but also the impaired glucose tolerance and insulin resistance. Treatment with ASW also significantly prevented an increase of the fasting insulin concentration, compared with the HFD group, while there was a small, similar effect with treatment with ASE. These data indicate that dietary intervention with 1% ASE and 3% ASW for ten weeks protected against the HFD-induced enhanced fasting glucose and insulin, as well as impaired glucose tolerance and insulin resistance.

### 3.2. ASE and ASW Attenuated Hyperlipidemia in HFD Mice

**Figure 2:**
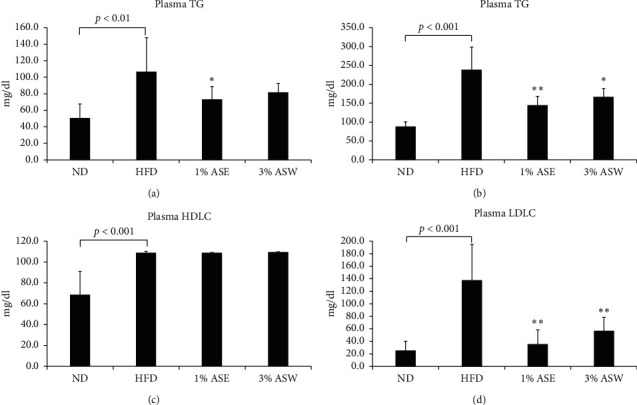
Effect of ASE and ASW on serum lipid levels in C57BL/6J mice fed by HFD. The levels of serum TG (a), TC (b), HDL-C (c), and non-HDL-C (d). Data are shown as means ± SEM (*n* = 6 in each group). HFD vs. ASE or ASW, ^*∗*^*p* < 0.05; ^*∗∗*^*p* < 0.01.

Variation of the lipid composition of the serum is one of the features of metabolic syndrome [22]. Therefore, serum TG, TC, HDLC, and LDLC levels were monitored to evaluate the effect of ASE and ASW on lipid metabolism. We found that feeding HFD for 10 weeks significantly increased the levels of serum TG, TC, and LDLC, leading mice to hyperlipidemia. Interestingly, the increase in the level of serum TG was significantly prevented in the 1% ASE group (Figure 2(a)), and the increases in the levels of serum TC and LDLC were prevented significantly in the 1% ASE and 3% ASW groups (Figures 2(b) and 2(d)). The level of serum HDLC did not differ significantly from the HFD group after ASE or ASW treatment (Figure 2(c)). These results indicate that intervention with ASE or ASW inhibited hyperlipidemia in HFD mice.

### 3.3. ASE and ASW Alleviated Hepatic Steatosis and Hepatic Inflammation in HFD Mice

**Figure 3:**
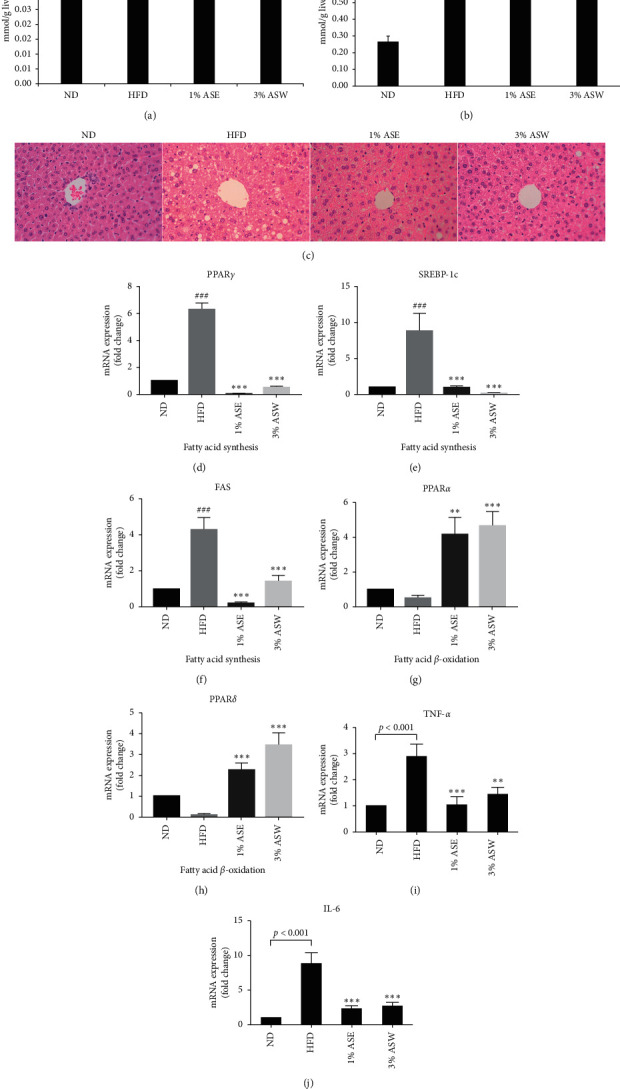
Effect of ASE and ASW on hepatic steatosis and inflammation in the livers of C57BL/6J mice fed by HFD. (a) Changes in hepatic TG. (b) Changes in hepatic TC. (c) Hematoxylin and eosin staining of transverse liver sections (original magnification ×200). (d–f) The hepatic de novo lipogenesis-related gene expression. (d) PPAR*γ*. (e) SREBP-1c. (f) FAS. (g and h) The hepatic *β*-oxidation-related gene expression. (g) PPAR*α*. (h) PPAR*δ*. (i) Hepatic inflammation factor, TNF-*α*. (j) Hepatic inflammation factor, IL-6. Data are shown as means ± SEM (*n* = 6 in each group). HFD vs. ASE or ASW, ^*∗∗*^*p* < 0.01; ^*∗∗∗*^*p* < 0.001.

The liver, as a central regulator of lipid homeostasis, is an essential organ in lipid metabolism [23]. An imbalance of lipid metabolism is related to metabolic dysfunctions and may precipitate the retention of fat within the liver and the subsequent development of NAFLD [24]. We investigated the effect of administration of ASE or ASW on treatment of NAFLD in HFD mice. Feeding HFD for 10 weeks resulted in a significantly (31.4%) higher hepatic TG content than the ND group, while supplementation with 1% ASE or 3% ASW led to only 19.3% and 4.1% higher hepatic TG content than the ND group, respectively (Figure 3(a)), indicating that ASE and ASW significantly prevented lipid deposition in HFD mouse livers. There was no difference among the groups in the amount of hepatic cholesterol (Figure 3(b)). H&E staining revealed that a significant amount of lipid droplet accumulated in the hepatocytes of HFD mice, compared with ND mice, but this symptom of NAFLD was prevented by ASE or ASW supplementation for 10 weeks (Figure 3(c)). Moreover, to examine the effect of ASE and ASW on hepatic steatosis in the HFD mice liver, we identified the changes of genes associated with fatty acid synthesis and *β*-oxidation. The expression of genes involved in de novo lipogenesis in hepatocytes, peroxisome proliferator-activated receptor gamma (PPAR*γ*), sterol regulatory element-binding protein 1 (SREBP1), and fatty acid synthase (FAS) was substantially higher in the HFD group than the ND group, while all the genes were expressed at greatly lower levels after treatment with ASE and ASW, compared with the HFD group (Figures 3(d)–3(f)). The expression of transcription factors, peroxisome proliferator-activated receptor alpha (PPAR*α*), and peroxisome proliferator-activated receptor delta (PPAR*δ*), associated to fatty acid *β*-oxidation, was markedly increased by treatment with ASE and ASW, compared with the HFD group (Figures 3(g) and 3(h)). The data indicate that ASE and ASW treatment leads to upregulation of fatty acid oxidation and downregulation of fatty acid synthesis, resulting in the amelioration of liver steatosis. To further examine the level of inflammation in the mice liver, the inflammatory factors TNF-*α* and interleukin 6 (IL-6) were analyzed. The results showed that both TNF-*α* and IL-6 in the liver tissue of the high-fat diet group were higher than those of the normal diet group, indicating the high-fat diet induced liver inflammation, while treating with 1% ASE or 3% ASW significantly decreased the inflammatory factors TNF-*α* and IL-6, preventing the liver in an inflamed state (Figures 3(i) and 3(j)). Taken together, treatment with ASE or ASW prevented hepatic steatosis in HFD mice.

### 3.4. ASE and ASW Improved Liver Function in HFD Mice

**Figure 4:**
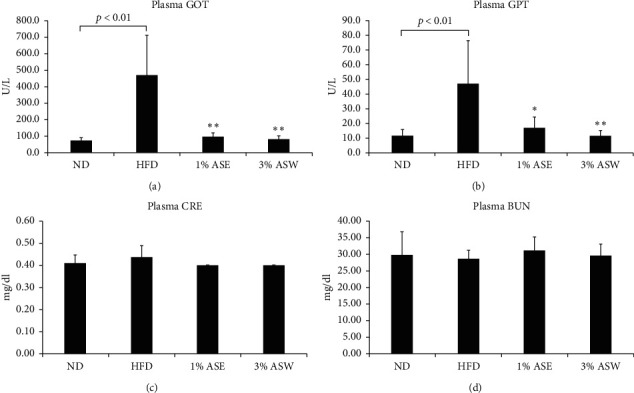
Effect of ASE and ASW on the serum levels of hepatic steatosis-related markers in C57BL/6J mice fed by HFD. (a and b) The serum levels of the hepatic lipotoxicity markers GOT and GPT. (c and d) The serum levels of the kidney toxicity markers CRE and BUN. Data are shown as means ± SEM (*n* = 6 in each group). HFD vs. ASE or ASW, ^*∗*^*p* < 0.05; ^*∗∗*^*p* < 0.01.

Excess lipid accumulation in the liver has a strong association with insulin resistance and low-grade hepatic inflammation, which may cause NAFLD to develop to the more severe form, NASH [25]. Here, we found that the serum GOT and GPT levels of HFD mice were significantly higher than those of ND mice, showing that the liver was in an injured state and that was significantly prevented after 10 weeks of feeding 1% ASE or 3% ASW (Figures 4(a) and 4(b)). Besides, neither the group which fed HFD nor the groups which fed ASE or ASW experienced a significant change in the levels of serum creatinine (CRE) and blood urea nitrogen (BUN), which are commonly used as markers of kidney function in the clinic (Figures 4(c) and 4(d)). These data indicate that ASE and ASW supplementation improved liver function during HFD-induced NAFLD development.

### 3.5. ASE and ASW Inhibited the Hepatic de novo Lipogenesis Pathway and Induced Fatty Acid *β*-Oxidation in a Human Fatty Liver Cell Model

**Figure 5:**
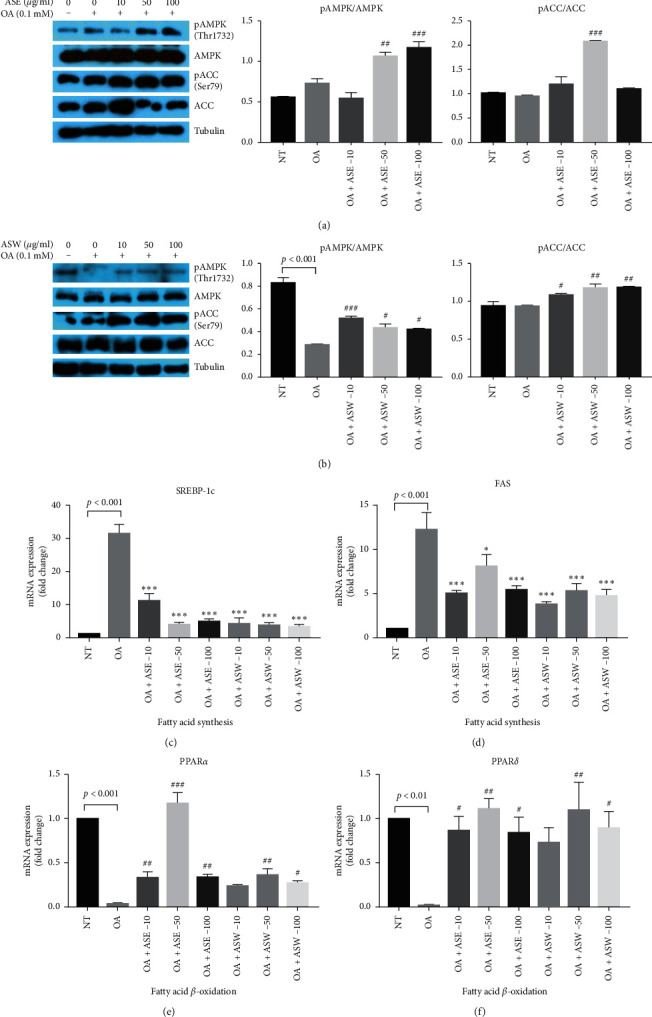
The mechanism of ASE and ASW on improving hepatic steatosis in OA-treated HuS-E/2 cells. (a and b) The protein factors related to hepatic de novo lipogenesis and fatty acid *β*-oxidation pathway were analyzed by western blotting. The protein expression of phosphorylation of AMPK at Thr172 and ACC at Ser79, total AMPK, and ACC under the condition of ASE treatment are shown in panel (a). Those protein expressions under the condition of ASW treatment are shown in panel (b). Tubulin served as a loading control. Quantitative analysis with Multi Gauge V3.0 is shown. (c–f) The gene expression related to hepatic de novo lipogenesis and fatty acid *β*-oxidation pathway is analyzed by RT-qPCR. The expressions of fatty acid synthesis-related genes: (c) FAS and (d) SREBP-1c. The expression of fatty acid *β*-oxidation-related genes: (e) PPAR*α* and (f) PPAR*δ*. Experiments are performed in triplicate, and data are shown as means ± SEM. OA vs. ASE or ASW, ^#^*p* < 0.05; ^##^*p* < 0.01; ^###^*p* < 0.001 in increased level. OA vs. ASE or ASW, ^*∗*^*p* < 0.05; ^*∗∗∗*^*p* < 0.001 in decreased level.

An increase in intrahepatic TG (IHTG) content is the hallmark characteristic of NAFLD. In order to determine the mechanism of the effect of ASE and ASW, hepatic de novo lipogenesis-related proteins and gene expression were examined in a human fatty liver cell model by using an immortalized human primary hepatocyte, HuS-E/2 cells [26]. AMPK plays a crucial role in regulation of fat metabolism in the liver, and the activation of AMPK phosphorylates its downstream target enzyme, ACC, by phosphorylation at Ser-79 [27]. We found that treatment with ASE or ASW significantly increased both pAMPK and pACC, respectively (Figures 5(a) and 5(b)). Also, treatment of OA led to higher gene expression of fatty acid synthase (FAS) and sterol regulatory element-binding protein-1c (SREBP-1c) than that of NT control, while treatment with ASE or ASW significantly lowered both FAS and SREBP-1c gene expressions, which are involved in fatty acid synthesis (Figures 5(c) and 5(d)). In addition, the gene expression of peroxisome proliferator-activated receptor alpha (PPAR*α*) and peroxisome proliferator-activated receptor delta (PPAR*δ*) in the OA treatment was lower than that of the NT control, while treatment of ASE and ASW significantly increased PPAR*α* and PPAR*δ* gene expressions, associated to fatty acid *β*-oxidation (Figures 5(e) and 5(f)). These data reveal that ASE and ASW inhibited the hepatic de novo lipogenesis pathway and promoted the fatty acid *β*-oxidation pathway.

## 4. Discussion

As one of the most common liver diseases, with increasing prevalence worldwide, NAFLD is a progressive pathological condition and is associated with many metabolic comorbidities, including obesity, type II diabetes, dyslipidemia, and metabolic syndrome, which promote the development of more severe liver disease, such as NASH, advanced fibrosis, cirrhosis, and hepatocellular carcinoma (HCC) [28]. To date, no pharmacotherapy targeting NAFLD is available, except for preventative lifestyle improvements and physical exercise to lose weight [29]. Novel treatments for NAFLD are sought urgently.

Adlay is an annual herb planted widely in Taiwan, China, and Japan, and its seeds have served as a dietary supplement and traditional Chinese medicine to treat warts, chapped skin, rheumatism, and neuralgia for thousands of years [12]. Apart from the ethnopharmacological function, several bioactive ingredients in adlay seeds have been isolated and identified, such as coixenolide, lactams, coixol, polysaccharides, plant sterols, and triterpenes [30, 31], which possess antiproliferative, antitumor, and various immunomodulatory activities for treating cancer, metastasis, hypertension, arthritis, asthma, and immunological disorders [32, 33]. The pharmacokinetics of adlay seeds have been investigated *in vivo*. Moreover, adlay seeds are considered safe for routine oral administration and external use and reduce the side effects of chemotherapy [34]. Among several kinds of extraction methods, the water and ethanolic extracts of adlay seeds are well known for their bioactive properties. The water extract of adlay seeds was reported to contain coixans A, B, and C and possess hypoglycemia activity [15]. The ethanolic extracts of adlay seeds rich in ferulic acid possess activities for adipose differentiation [35]. However, few studies have investigated the treatment effects of adlay seed extracts on NAFLD. Therefore, we consider the extracts of adlay seeds may provide a novel strategy with great potential for the treatment of NAFLD.

One of the common comorbidities of NAFLD is dyslipidemia [36], and studies have shown that NAFLD and dyslipidemia are strongly related [37, 38]. To determine the effect of extracts of adlay seeds on mice with diet-induced metabolic dysfunction, we mixed 1% ASE or 3% ASW, respectively, with HFD as the feed for mice for 10 weeks. We found that treatment with either ASE or ASW for 10 weeks prevented the higher levels of fasting blood glucose, insulin, glucose tolerance, and HOMA-IR caused by feeding HFD to mice. Furthermore, dyslipidemia was observed in the HFD mice, and supplementation with ASE or ASW significantly ameliorated the increases in the concentrations of serum TC and LDLC, and treatment with ASE significantly prevented increases in the concentrations of serum TG. Recent studies investigated NAFLD, as well as atherosclerosis, resulting from hypercholesterolemia [39, 40]. Our result showed that ASE and ASE improved hypercholesterolemia of HFD mice, which is consistent with a previous study [17], suggesting the potential treatment effect on not only NAFLD but also atherosclerosis.

NAFLD is thought to be a leading cause of abnormal liver function [1, 2]. We confirmed that feeding HFD for 10 weeks indeed induced the development of early lesions of NAFLD in the livers of mice, while the supplementation with ASE or ASW significantly prevented increases not only in the levels of hepatic TG but also the accumulation of lipid droplets within hepatocytes. Lipotoxicity is a harmful effect of lipid accumulation in nonadipose tissue, leading to liver inflammation and fibrosis [41]. To investigate the hepatic inflammation, we tested the crucial inflammatory factors, TNF-*α* and IL-6, as well as the clinical parameters, GOT and GPT, as markers of liver injury [42]. Increased gene expression of TNF-*α* and IL-6 and the levels of serum GOT and GPT by feeding HFD for 10 weeks were significantly attenuated by ASE or ASW supplementation, indicating improvement preventative effect of ASE and ASW on hepatic inflammation and injury. In addition, no significant differences in serum CRE and BUN were observed among the groups, showing no influence of ASE and ASW on kidney function.

We also verified the mechanism of the effect of ASE and ASW. In our study, we used the HuS-E/2 cell line, which was derived from human primary hepatocytes and has been shown to be phenotypically and functionally similar to human primary hepatocytes [21, 43]. To unveil the underlying mechanism of the effect of ASE and ASW on ameliorating NAFLD in high-fat diet-induced mice, we simulated a high-fat environment in the HuS-E/2 cell line and treated with different doses of ASE or ASW. Our results showed ASE and ASW significantly increased the protein expression of pAMPK and pACC. One of the identified AMPK targets is ACC, playing a role in the control of fatty acid metabolism via the regulation of malonyl-CoA synthesis. By phosphorylation to inhibit ACC and lowering the concentration of its reaction product malonyl-CoA, AMPK activation is expected to coordinate the partitioning of fatty acids between oxidative and biosynthetic pathways by increasing fatty acid oxidation capacity and inhibiting de novo lipogenesis [44]. Also, treatment with ASE or ASW significantly decreased the gene expression of FAS and SREBP-1c and significantly increased the gene expression of PPAR*α* and PPAR*δ*. SREBP-1c activates other lipogenic genes, such as ACC and FAS, and prevents fatty acids from *β*-oxidation [45]. PPAR*α* and *δ* are correlated to mitochondrial fatty acid *β*-oxidation and lead fatty acid to go into the mitochondrial and to be metabolized [46].

Despite the results in our study which indicate the prophylactic effect of adlay seed extracts on improving NAFLD, there was still limitation in our study. NAFLD with hepatic necroinflammation and faster progression to fibrosis may develop into NASH, and even to HCC [47]. Whether ASE and ASW have therapeutic effect on NASH should be further investigated.

## 5. Conclusions

In conclusion, our study indicated that ASE and ASW ameliorated the symptoms of hyperglycemia and impaired glucose tolerance, hyperlipidemia, hepatic steatosis, and hepatic inflammation via inhibiting hepatic de novo lipogenesis and promoting fatty acid *β*-oxidation. These results demonstrated the potential therapeutic effect of ASE and ASW on NAFLD and diet-induced metabolic dysfunction.

## Figures and Tables

**Table 1:** Biological parameters of mice after 10 weeks of treatment.

Parameter	HFD	HFD-1% ASE	HFD-3% ASW	ND
Body weight (g)	35.90 ± 1.04	37.40 ± 2.72	34.78 ± 1.73	26.85 ± 0.72^*∗∗∗*^
Food intake (g)	3.92 ± 0.68	3.95 ± 0.90	3.99 ± 2.66	3.59 ± 0.49
Adipose tissue weight (g)	2.16 ± 0.16	2.02 ± 0.44	2.11 ± 0.23	0.33 ± 0.07^*∗∗∗*^
Liver weight (g)	1.11 ± 0.05	1.18 ± 0.15	1.02 ± 0.06^*∗∗*^	0.93 ± 0.07^*∗∗∗*^

(a) ND, normal diet; HFD, high-fat diet; ASE, ethanolic extract of adlay seeds; ASW, water extract of adlay seeds. (b) All data are shown as means ± SEM, *n* = 8 per group. Data of different groups were compared with the corresponding data from HFD-fed mice. Differences were examined for statistical significance using Student's *t*-test. (c) HFD vs. adlay extracts and ND: ^*∗∗*^*p* < 0.01 and ^*∗∗∗*^*p* < 0.001 indicated decreased level.

## Data Availability

The data used to support the findings of this study are available from the corresponding author upon request.
